# Supplementary material for: Reverse C-glycosidase reaction provides C-nucleotide building blocks of xenobiotic nucleic acids
Source: Nat Commun. 2020 Dec 8;11:6270. doi: 10.1038/s41467-020-20035-0 (PMC7722734; doi:10.1038/s41467-020-20035-0)
Supplement: Supplementary file 1 — Supplementary Information [file 41467_2020_20035_MOESM1_ESM.pdf]

Supplementary Information

Reverse *C*-Glycosidase Reaction Provides *C*-Nucleotide Building Blocks of Xenobiotic Nucleic Acids

Martin Pfeiffer[1,2], Bernd Nidetzky[1,2,*]

[1] Institute of Biotechnology and Biochemical Engineering, Graz University of Technology, NAWI Graz, Petersgasse 12, A-8010 Graz, Austria

[2] Austrian Centre of Industrial Biotechnology (acib), Petersgasse 14, A-8010 Graz, Austria

* Corresponding author; e-mail: bernd.nidetzky@tugraz.at; phone: +43 3168738400

Supplementary Figures 4
Supplementary Figure 1 *C*-Nucleosides with reported antiviral or antimicrobial efficacy 4
Supplementary Figure 2 YeiN catalyzed cleavage of the *C*-glycosidic bond of ΨMP 5
Supplementary Figure 3 Proposed mechanism of *C*-glycoside synthesis by AlnA 6
Supplementary Figure 4 SDS polyacrylamide gel … 7
Supplementary Figure 5 HPLC trace of reference substances 8
Supplementary Table 5 Kinetic parameters of YeiN 9
Supplementary Figure 6 TLC analysis of pentose phosphate formation 10
Supplementary Figure 7 Substrate scope of RbsK 11
Supplementary Figure 8 Substrate specificity of YeiN analyzed with molecular docking of sugar phosphates 12
Supplementary Figure 9 Crystal structure of covalently linked ΨMP intermediate (yellow; pdb: 4GIL) 13
Supplementary Figure 10 Substrate specificity of YeiN analyzed by molecular docking of nucleobase analogues into the activesite of YeiN involving a covalently linked Rib5P (cyan) intermediate (iminium form) 14
Supplementary Figure 11 ^{1}H NMR analysis of YeiN catalyzed deuterium incorporation into the C5 of Ura 15
Supplementary Figure 12 Time courses YeiN catalyzed synthesis of ΨMP derivatives 16
Supplementary Figure 13 Time course of the ΨMP dephosphorylation using calf intestine phosphatase 17
Supplementary Table 6 Substrate scope of YeiN 18
Supplementary Figure 14 Comparison of ΨMP phosphorylation with and without NDK 19
Supplementary Figure 15 OPMEC synthesis of a) 6-amino-ΨTP and b) Ara-ΨTP 20
Supplementary Figure 16 Product isolation 21
Supplementary Figure 17 ^{1}H NMR of ΨMP 22
Supplementary Figure 18 ^{13}C NMR of ΨMP 22
Supplementary Figure 19 ^{1}H NMR of dΨMP 23
Supplementary Figure 20 ^{13}C NMR of dΨMP 23
Supplementary Figure 21 ^{1}H NMR of Ara-ΨMP 24
Supplementary Figure 22 ^{13}C NMR of Ara-ΨMP 24
Supplementary Figure 23 ^{1}H NMR of 4-thio-ΨMP 25
Supplementary Figure 24 ^{13} NMR of 4-thio-ΨMP 25
Supplementary Figure 25 ^{1}H NMR of 2-thio-ΨMP 26
Supplementary Figure 26 ^{13}C NMR of 2-thio-ΨMP 26
Supplementary Figure 27 ^{1}H NMR of 6-amino-ΨMP 27
Supplementary Figure 28 ^{12}C NMR of 6-amino-ΨMP 27
Supplementary Figure 29 ^{1}H NMR of 3-methyl-ΨMP 28

Supplementary Figure 30 ^{13}C NMR of 3-methyl-ΨMP 28
Supplementary Figure 31 ^{1}H NMR of Ψ 29
Supplementary Figure 32 ^{13}C NMR of Ψ 29
Supplementary Figure 33 ^{1}H NMR of ΨTP 30
Supplementary Figure 34 ^{13}C NMR of ΨTP 30
Supplementary Figure 35 ^{1}H NMR of 2-deoxy-2-fluoro-ΨMP 31
Supplementary Figure 36 ^{13}C NMR of 2-deoxy-2-fluoro-ΨMP 31
Supplementary References **32**

Supplementary Figures

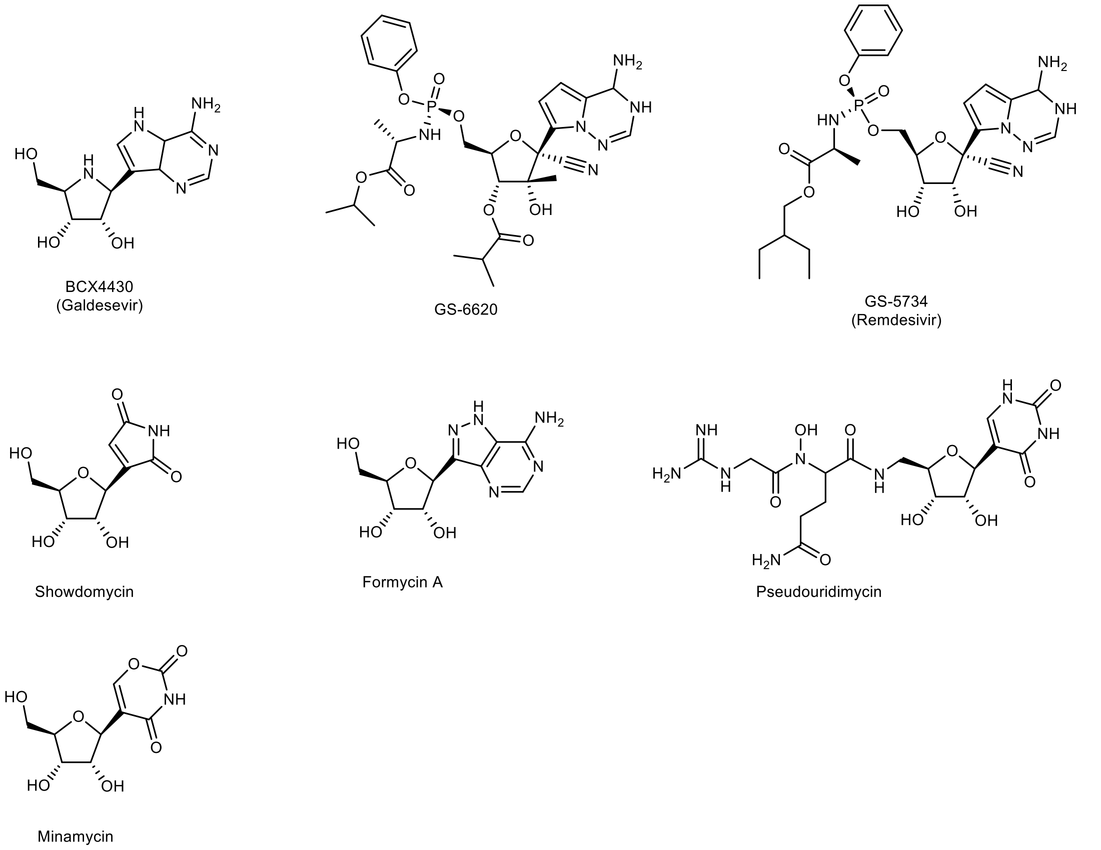

Supplementary Figure 1. *C*-Nucleosides with reported antiviral or antimicrobial efficacy.

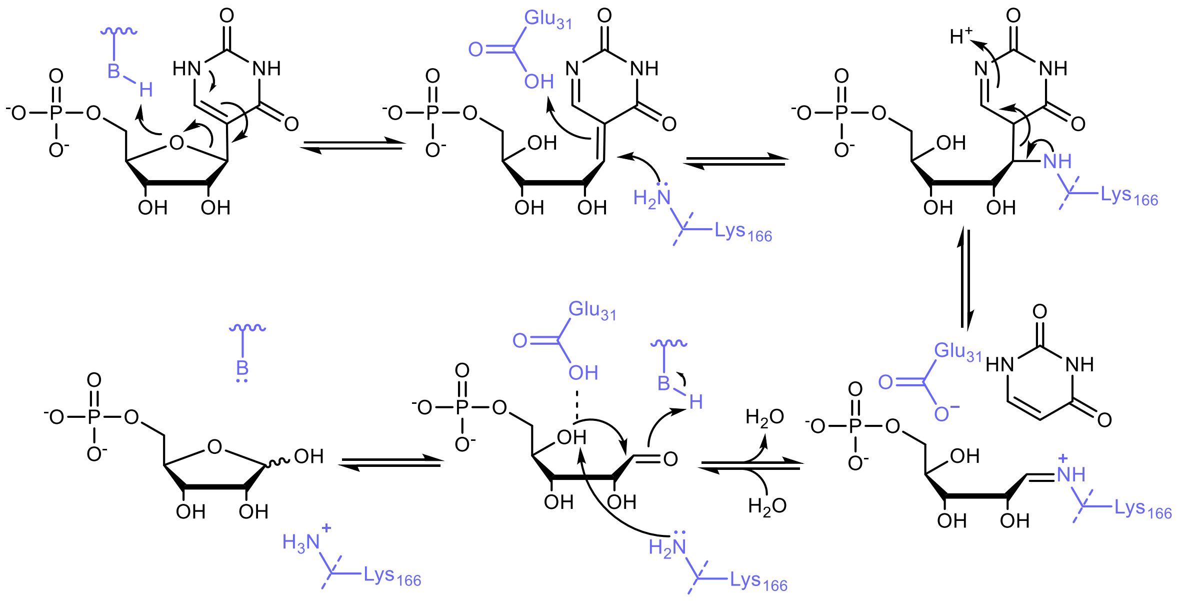

Supplementary Figure 2. YeiN-catalyzed cleavage of the *C*-glycosidic bond of ΨMP.[1]

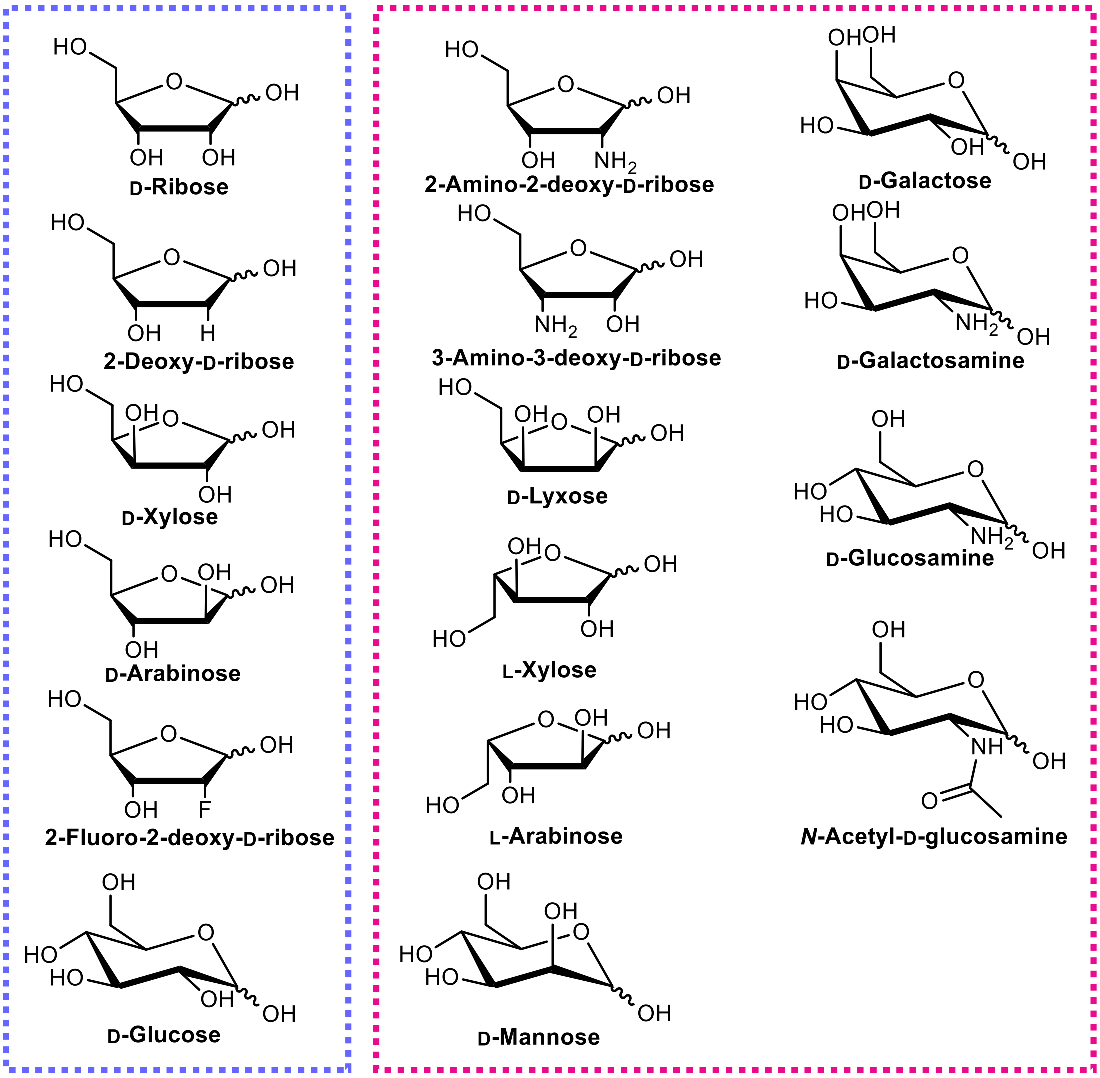

Supplementary Figure 3. Substrate scope of RbsK. Sugars framed in blue are active while those framed in magenta are not.

Supplementary Table 1. Substrate scope of YeiN evaluated in a combinatorial analysis.

	Rib5P Yield (%)[a]	dRib5P Yield (%)[a]	Ara5P Yield (%)[a]	Xyl5P Yield (%)[a]	2F-Rib5P Yield (%)[a]
Ura	99	90	80	30	15
Guanosine	n.a.	n.a.	n.a.	n.a.	n.a.
Adenine	n.a.	n.a.	n.a.	n.a.	n.a.
Cytosine	n.a.	n.a.	n.a.	n.a.	n.a.
Thymine	n.a.	n.a.	n.a.	n.a.	n.a.
Pyrimidine	n.a.	n.a.	n.a.	n.a.	n.a.
4-Pyrimidone	n.a.	n.a.	n.a.	n.a.	n.a.
Di-thio-Ura	n.a.	n.a.	n.a.	n.a.	n.a.
2-Thio-Ura	50	4	16	7	n.a.
4-Thio-Ura	99	84	40	18	n.a.
6-Trifluoromethyl-Ura	n.a.	n.a.	n.a.	n.a.	n.a.
6-Amino-Ura	80	10	6		n.a.
3-Methyl-Ura	99	65	26		n.a.
1-Methyl-Ura	n.a.	n.a.	n.a.	n.a.	n.a.
6-Aza-Ura	n.a.	n.a.	n.a.	n.a.	n.a.
6-Chloro-Ura	n.a.	n.a.	n.a.	n.a.	n.a.
5-Fluoro-Ura	n.a.	n.a.	n.a.	n.a.	n.a.
5-Ethyl-Ura	n.a.	n.a.	n.a.	n.a.	n.a.

[a]Yield of ΨMP or ΨMP-derivative after 3 h incubation based on the initial nucleobase concentration used. Reactions involved 5 mM pentose 5-phosphate, 1 mM nucleobase in 50 mM HEPES buffer (pH 7.0) supplemented with 2 mM $Mn2^{+}$ and 30 µM of YeiN at 37°C. For further experimental details and for the analytical procedures used, see the Methods section.

n.a., no activity detected.

Supplementary Table 2. Kinetic parameters of YeiN recorded at 37°C and pH 7.00.

Varied substrate	k_{cat} (s^{-1})	K_M (mM)	k_{cat}/K_M (s^{-1} mM^{-1})
Rib5P[1]	2.40 ± 0.17	0.36 ± 0.09	6.67 ± 0.47
dRib5P[1]	0.19 ± 0.01	0.54 ± 0.08	0.36 ±0.05
Ara5P[1]	0.03 ±0.01	0.30 ± 0.02	0.10 ±0.03
Xyl5P[1]	0.01 ±0.01	0.34 ± 0.04	0.03 ±0.1
2-fluoro-Rib5P[2]	~0.01		
Ura[3]	2.30 ±0.16	0.50 ± 0.04	4.60 ±0.32
6-amino-Ura[3]	0.01 ± 0.01	3.23 ± 0.52	0.01 ±0.01
2-thio-Ura[3]	0.43 ±0.10	7.4 ± 2.2	0.06 ±0.01
4-thio-Ura[3]	0.01 ±0.01	1.98 ± 0.30	0.005 ±0.001
3-methyl-Ura[3]	0.46 ±0.04	1.25 ± 0.33	0.37 ±0.10

[1] Ura (5 mM) was used (*n* = 3). [2] Apparent k_{cat} determined at 1 mM substrate (*n* = 3). [3]Rib5P (5 mM) was used.

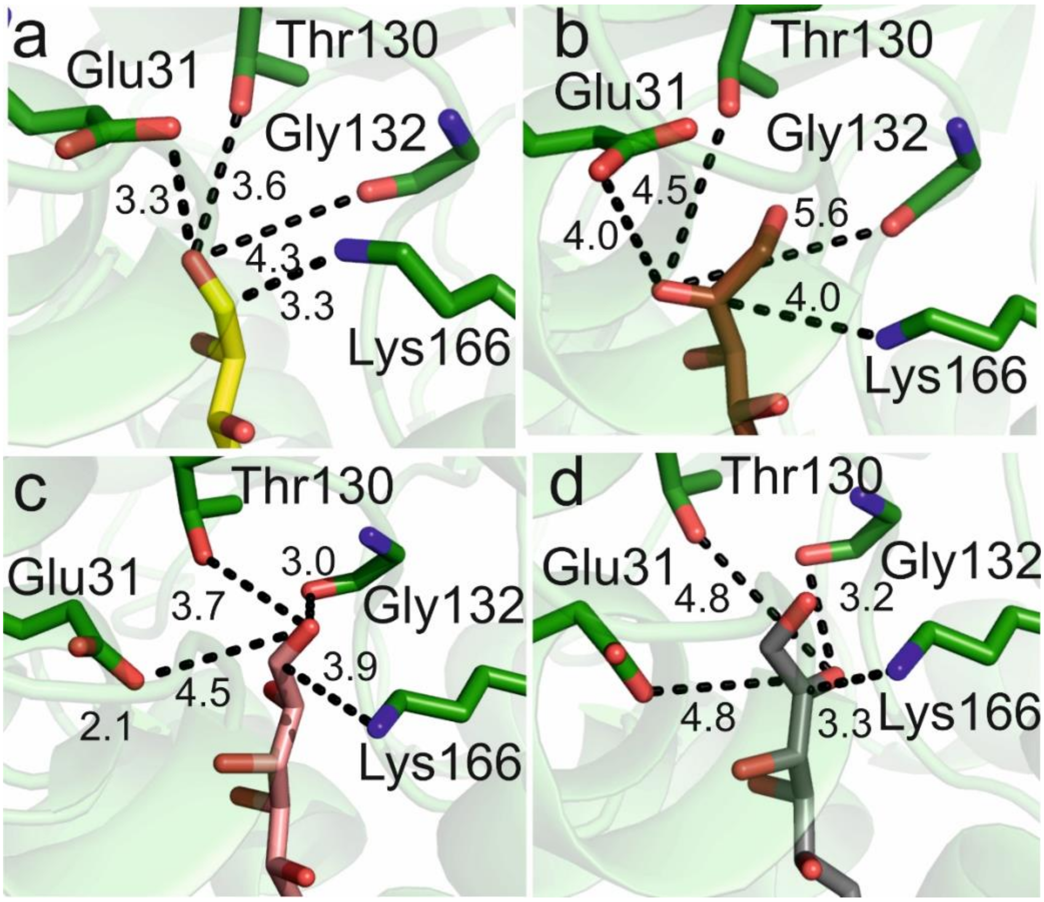

Supplementary Figure 4. Substrate specificity of YeiN analyzed with molecular docking of sugar phosphates. The YeiN structure (pdb code: 4GIL)[1] was used. Docking pose of a) Ery4P (**11**, yellow), b) Rul5P (**10**, brown), c) Glc6P (**7**, red), and d) Fru6P (**9**, gray). Catalytically relevant interactions are shown as dashed lines and distances are given in Å.

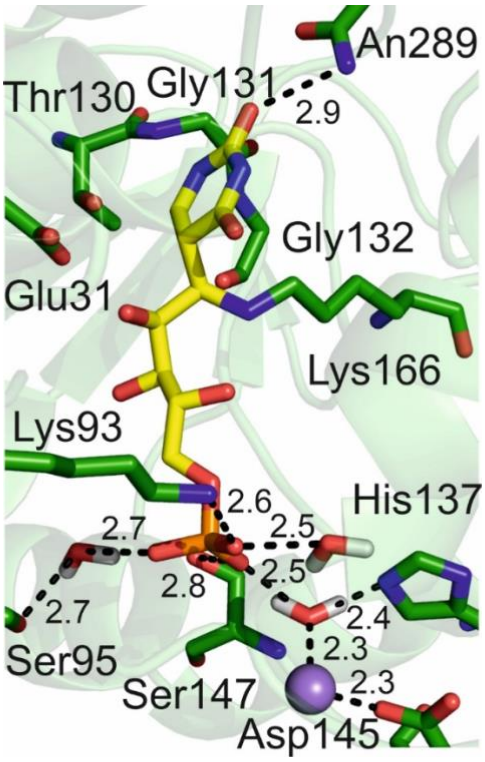

Supplementary Figure 5. Active-site close-up of the crystal structure (pdb: 4GIL[1]) of a covalent complex between YeiN and ΨMP intermediate. Enzyme interaction with phosphate and nucleobase are shown as dashed lines and distances are given in Å. Atom colouring is used. The C atoms of ΨMP are shown in yellow, those of YeiN in green. The enzyme-bound Mn^{2+} is shown as a violet ball.

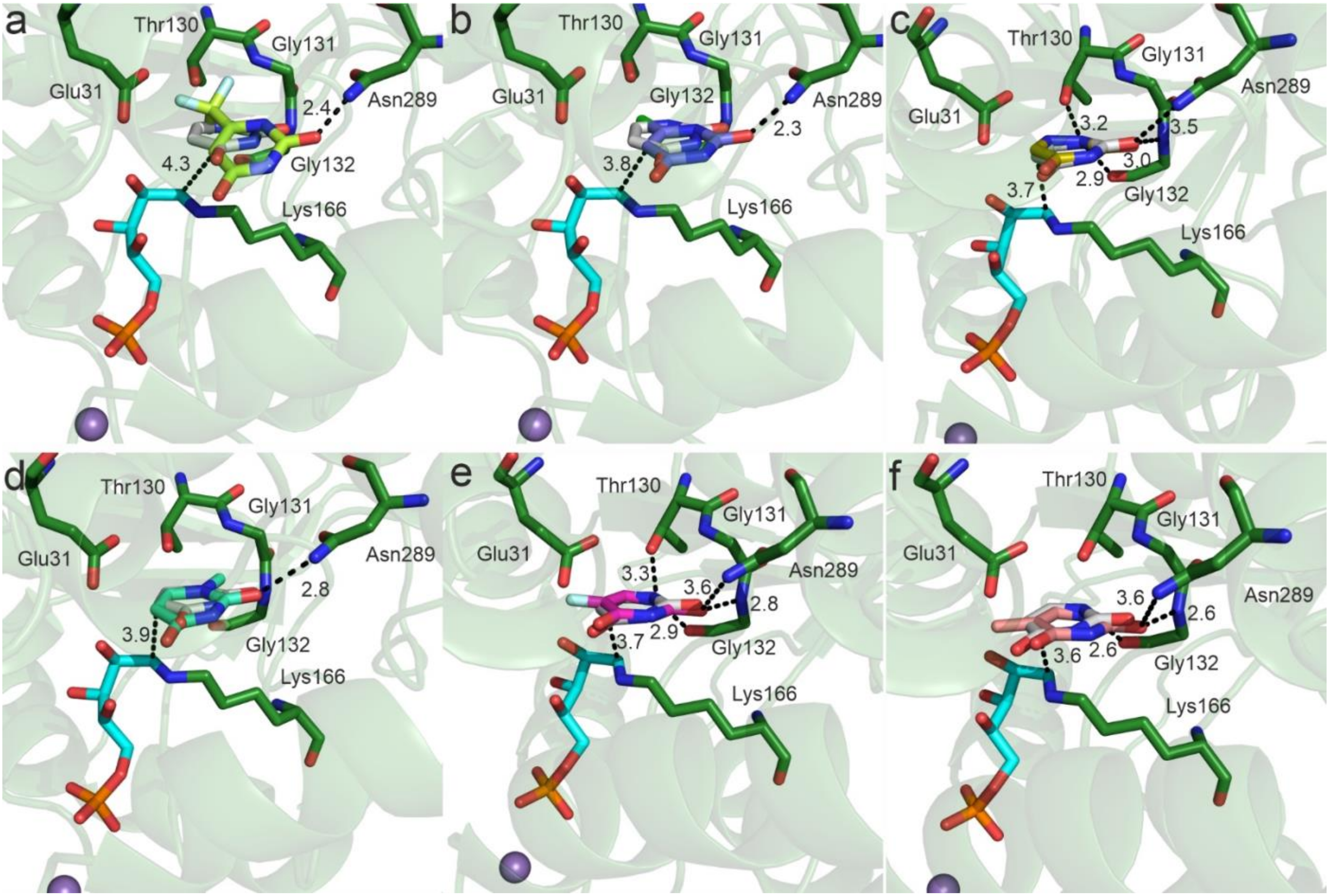

Supplementary Figure 6. The nucleobase substrate specificity of YeiN analyzed by molecular docking. A covalent iminium ion intermediate between the enzyme (Lys166) and the open-chain Rib5P was used as the protein receptor for ligand docking. Atom colouring is used. The C atoms of the enzyme and Rib5P are in green and cyan, respectively. The C atoms of the nucleobase ligands are coloured as indicated for each panel. In each panel, the docking poses for the relevant nucleobase ligand is superimposed on the docking pose of Ura (white). a) 6-trifluoromethyl-Ura (yellow); b) 6-Cl-Ura (blue); c) 6-aza-Ura (gold); d) 1-methyl-Ura (light green), e) 5-fluoro-Ura (purple); f) 5-methyl-Ura (light pink). Polar interactions and the distance between the Ura C5 and the Rib5P C1 are shown as dashed lines. Distances are depicted in Å. The enzyme-bound Mn^{2+} is shown as a violet ball.

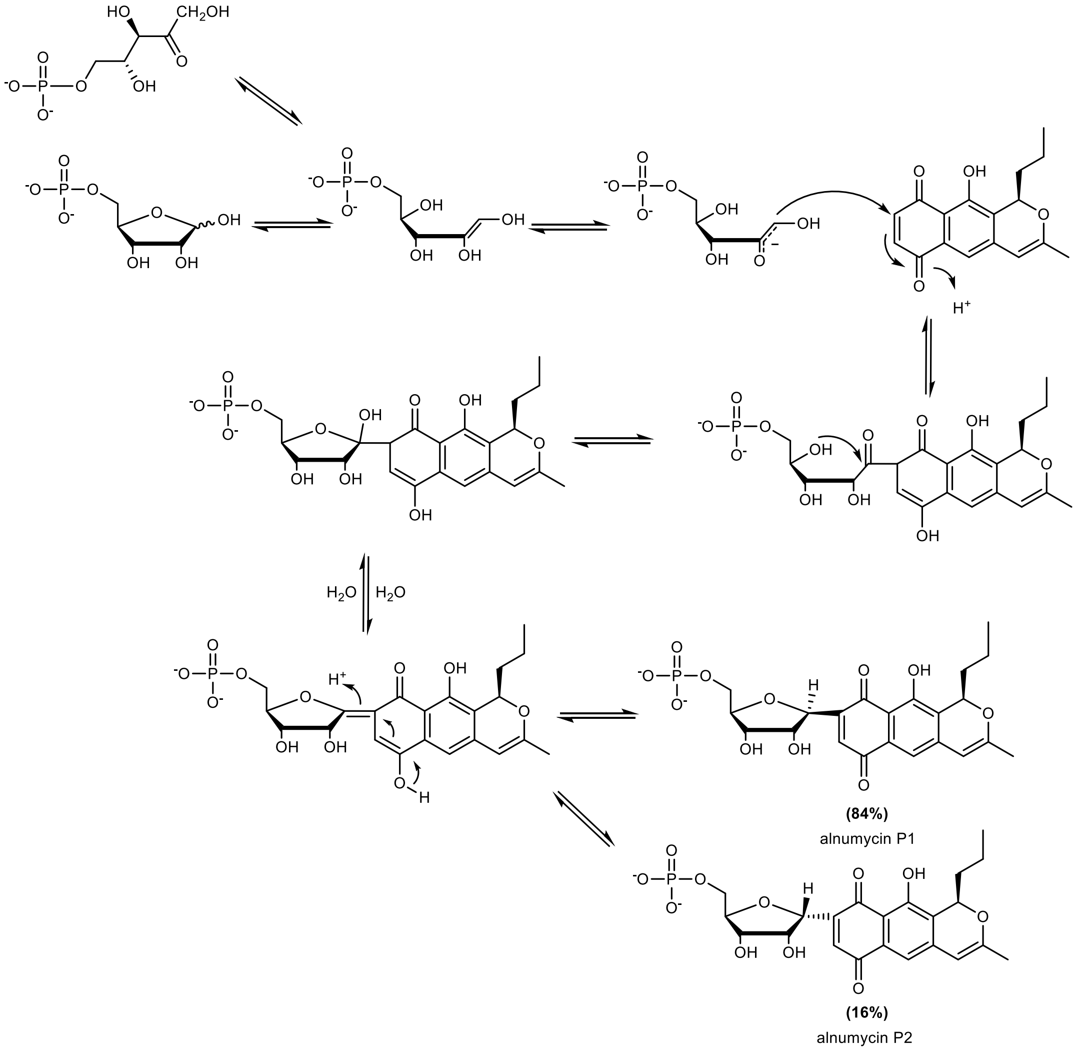

Supplementary Figure 7. Proposed mechanism of *C*-glycoside synthesis by AlnA. Note: ribose ring closure is suggested to occur without catalytic assistance by AlnA, resulting in the formation of both α- and β-configurated *C*-glycosidic products. [2,3]

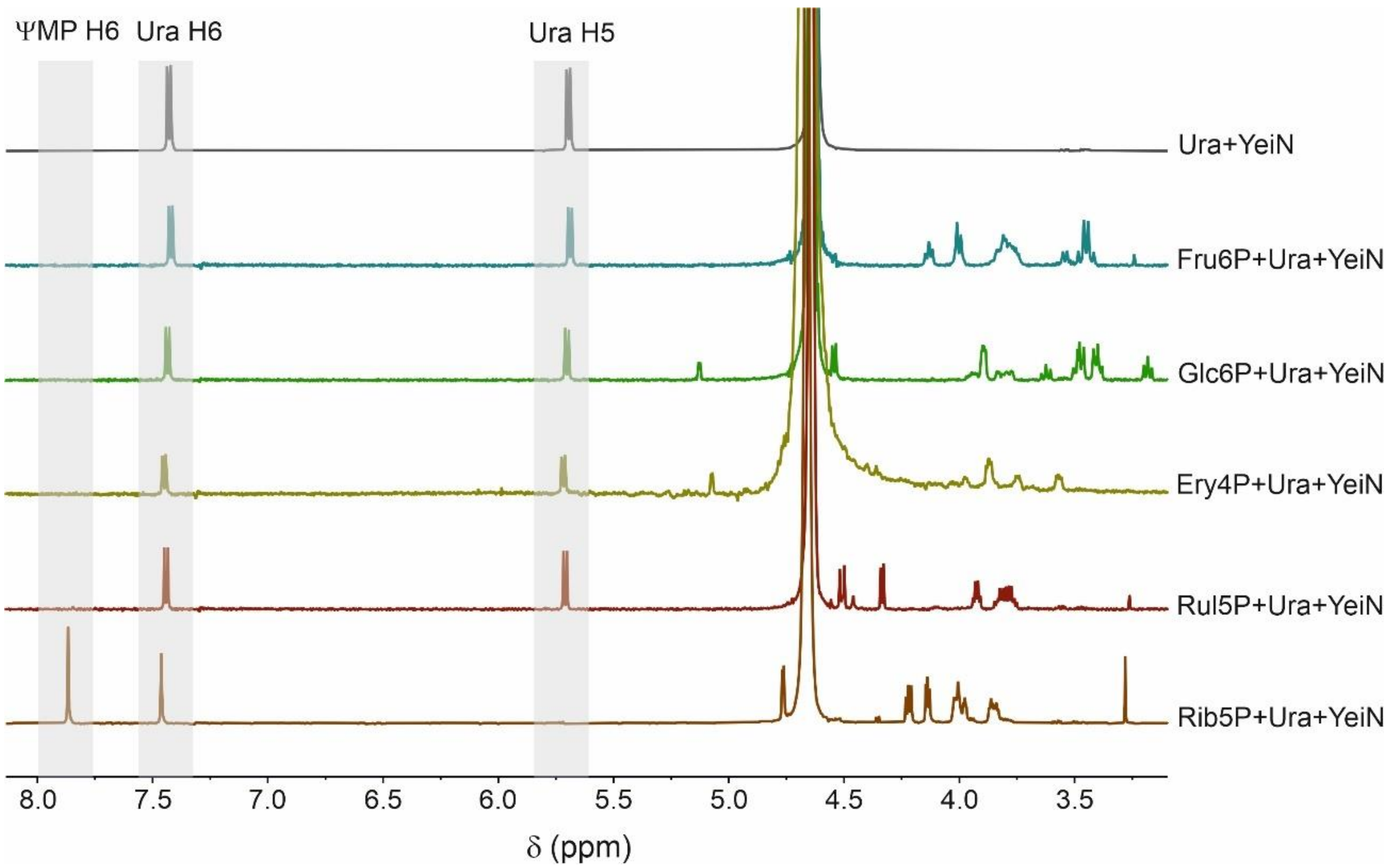

Supplementary Figure 8 ^{1}H NMR analysis of YeiN-catalyzed deuterium incorporation into the C5 of Ura. Reactions contained 3 µM YeiN, 5 mM Ura and either one of five tested sugar phosphates (5 mM Rib5P, Ery4P, Rul5P, Glc6P or Fru6P). Deuterium incorporation is only observed when transient formation of ΨMP occurs (*n* = 1).

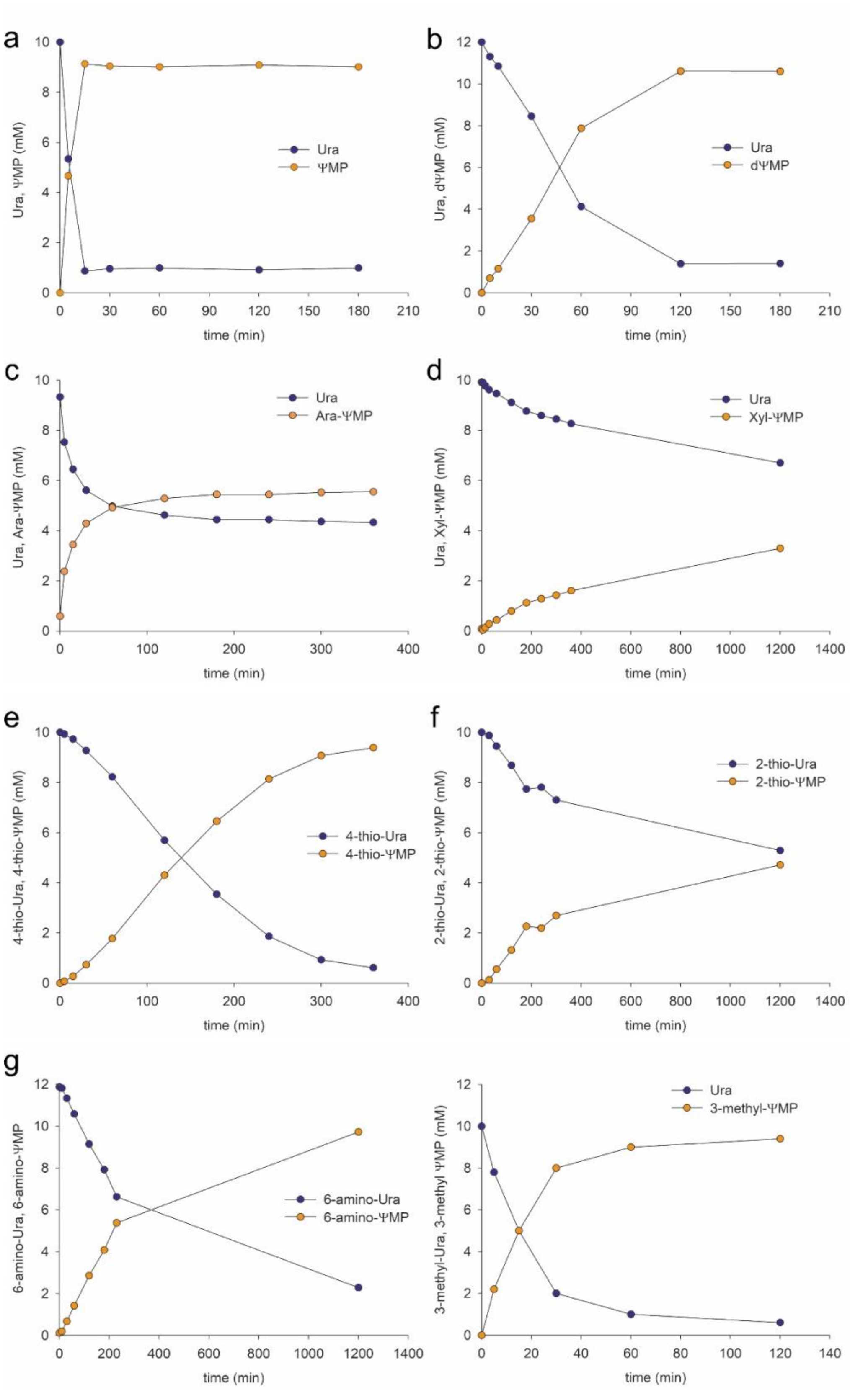

Supplementary Figure 9 Time courses of YeiN-catalyzed synthesis of ΨMP derivatives. a) ΨMP, b) dΨMP, c) 5-(β-D-arabinofuranosyl 5-phosphate)uracil (Ara-ΨMP), d) 5-(β-D-xylofuranosyl 5-phosphate)uracil (Xyl-ΨMP), e) 4-thio-ΨMP, f) 2-thio-ΨMP, g) 6-amino-ΨMP, h) 3-methyl-ΨMP are shown (all, *n* = 1). For further experimental details and for the analytical procedures used, see the Methods section.

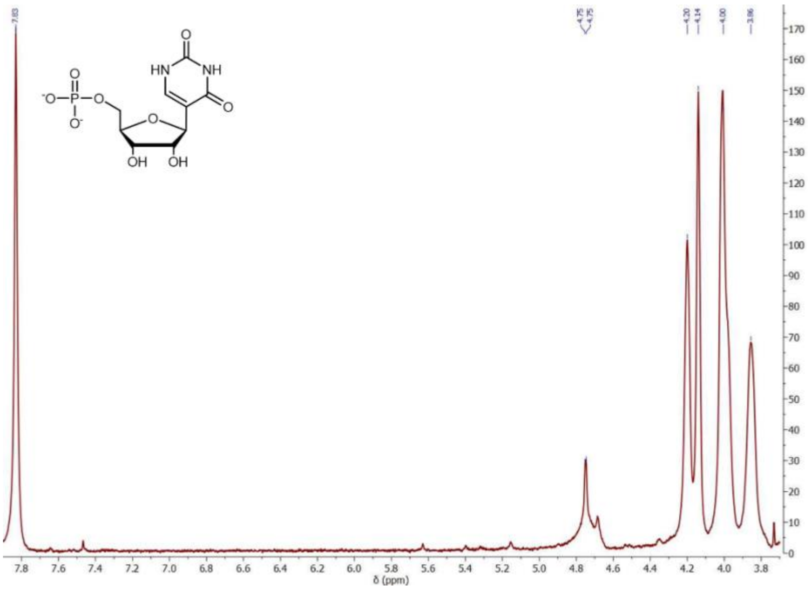

Supplementary Figure 10 ^{1}H NMR of ΨMP (500 MHz, D_2O): δ 7.83 (s, 1H, H-6), δ 4.75 (d, J = 2.8 Hz, 1H, H-1’), 4.20 (s, 1H, H-2’), 4.14 (s, 1H, H-3’), 4.01 (s, 1H, H-4’), 4.01 - 3.86 (s, H-5’).

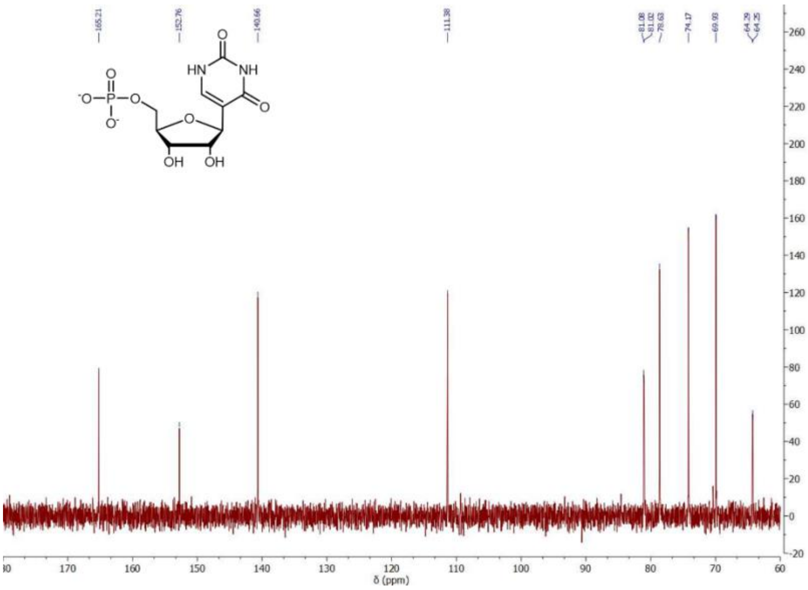

Supplementary Figure 11 ^{13}C NMR of ΨMP (126 MHz, D_2O): δ 165.21 (s, 1C, C-4), 152.77 (s, 1C, C-4), 140.67 (s, 1C, C-6), 111.38 (s, 1C, C-5), 81.02 (d, J = 8.5 Hz, 1C, C-4’), 78.64 (s, 1C, C-1’), 74.17 (s, 1C, C-2’), 69.93 (s, 1C, C-3’), 64.29 (d, J = 5.2 Hz, 1C, C-5’).

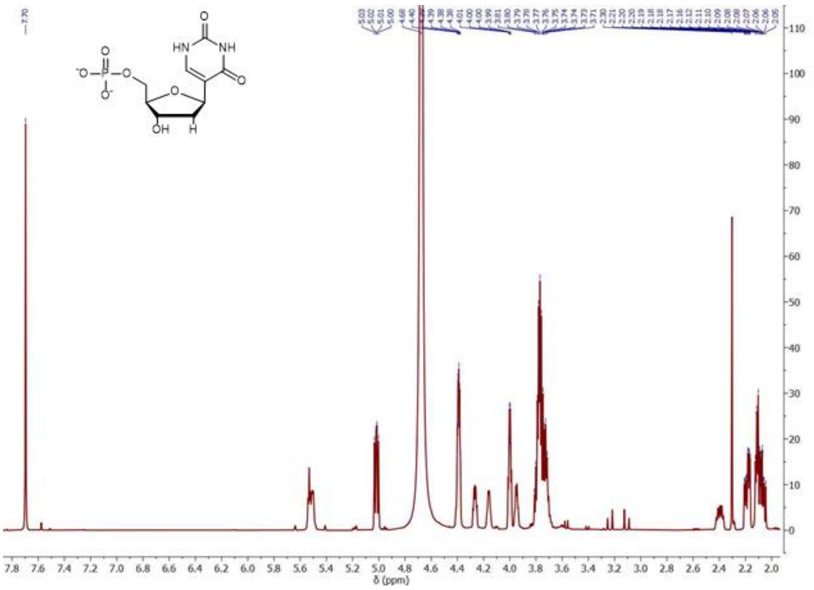

Supplementary Figure 12 ^{1}H NMR of dΨMP (500 MHz, D_2O): δ 7.70 (s, 1H, H-6), 5.02 (dd, J = 9.5, 6.2 Hz, 1H, H-1'), 4.39 (dd, J = 6.0, 3.1 Hz, 1H, H-3'), 4.00 (q, J = 2.0 Hz, 1H, H-4'), 3.80 – 3.75 (m, 2H, H-5'), 2.14 – 1.98 (m, 1H, H-2').

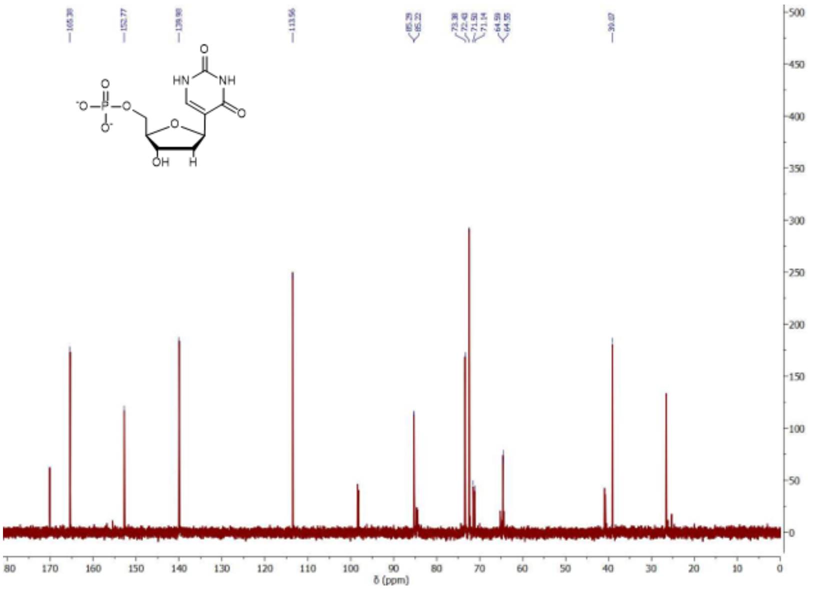

Supplementary Figure 13 ^{13}C NMR of dΨMP (126 MHz, D_2O): δ 165.38 (s, 1C, C-4), 152.77 (s, 1C, C-2), 139.98 (s, 1C, C-6), 113.56 (s, 1C, C-5), 85.26 (d, J = 8.4 Hz, 1C, C-4'), 73.38 (s, 1C, C-1'), 72.43 (s, 1C, C-3'), 64.57 (d, J = 4.8 Hz, 1C, C-5'), 39.07 (s, 1C, C-2').

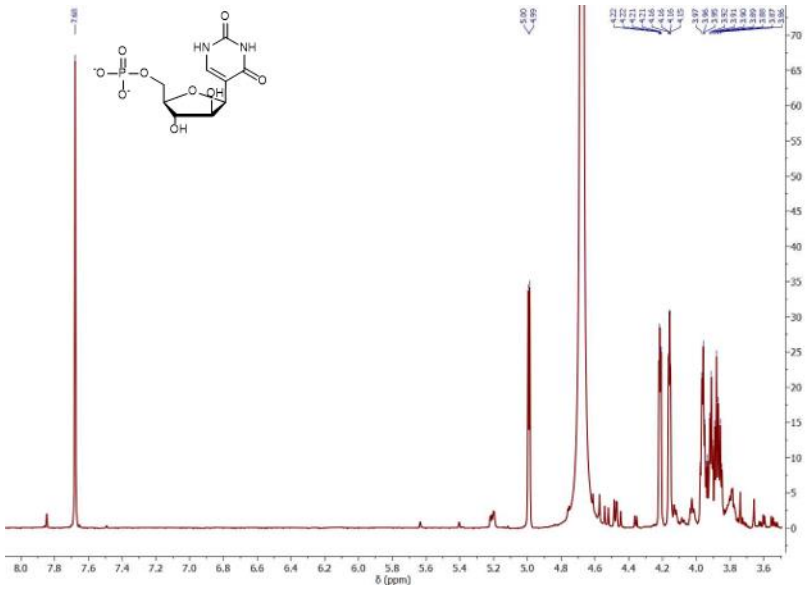

Supplementary Figure 14 ^{1}H NMR of 5-(β-D-arabinofuranosyl 5-phoshate)uracil (D-Ara-ΨMP) (500 MHz, D_2O): δ 7.68 (s, 1H, H-6), 4.99 (d, J = 4.3 Hz, 1H, H-1'), 4.21 (dd, J = 4.5, 2.3 Hz, 1H, H-2'), 4.16 (dd, J = 3.8, 2.4 Hz, 1H, H-3'), 3.99 – 3.84 (m, 3H, H-4' and H-5').

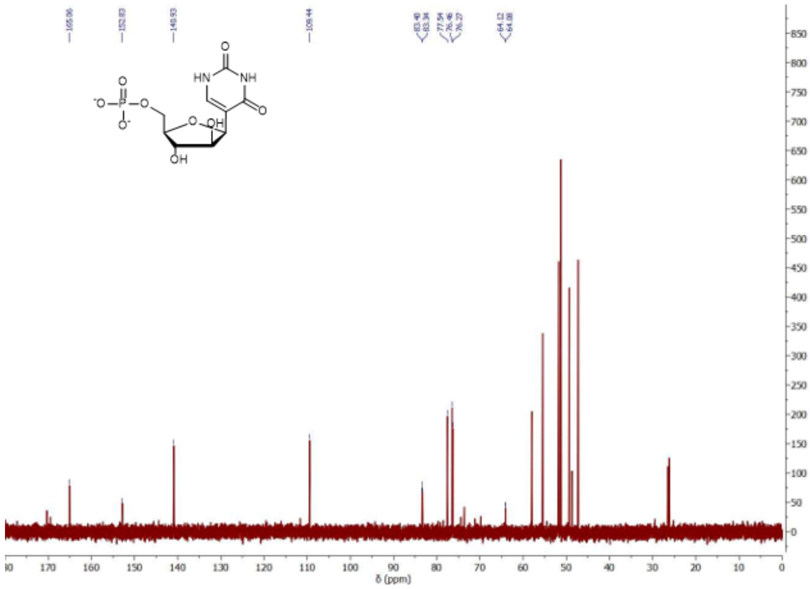

Supplementary Figure 15 ^{13}C NMR of 5-(β-D-arabinofuranosyl 5-phoshate)uracil (D-Ara-ΨMP) (126 MHz, D_2O): δ 165.06 (s, 1C, C-4), 152.83 (s, 1C, C-2), 140.93 (s, 1C, C-6), 109.44 (s, 1C, C-5), 83.37 (d, J = 8.4 Hz, 1C, C-4'), 77.54 (s, 1C, C-1'), 76.46 (s, 1C, C-2'), 76.27(s, 1C, C-3'), 64.10 (d, J = 4.4 Hz, 1C, C-5').

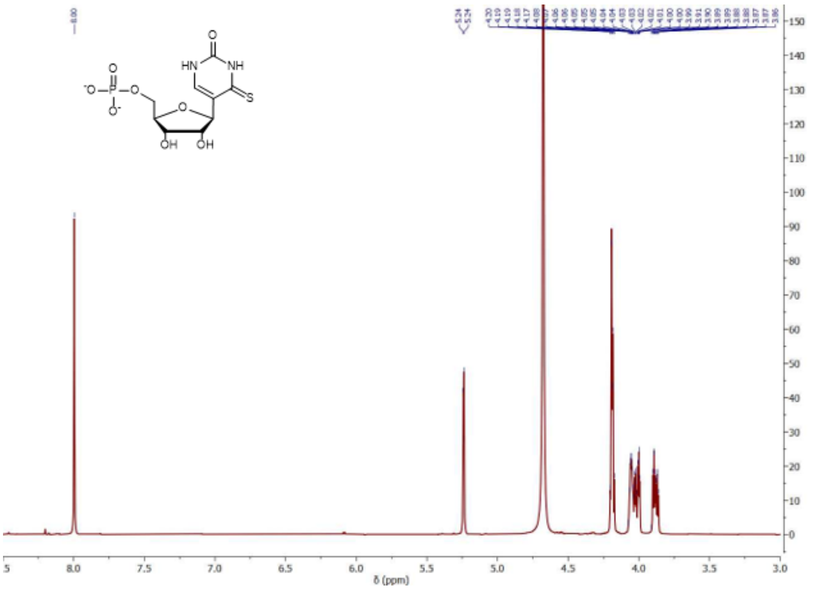

Supplementary Figure 16 ^{1}H NMR of 4-Thio-ΨMP (500 MHz, D_2O): δ 8.00 (s, 1H, H-6), 5.24 (d, J = 2.8 Hz, 1H, H-1’), 4.18 (dd, J = 6.3, 4.4 Hz, 2H, H3’), 4.08 – 4.04 (m, 1H, H-4’), 4.01 (ddd, J = 11.6, 4.4, 2.9 Hz, 1H, H-5’), 3.88 (ddd, J = 11.7, 5.1, 3.8 Hz, 1H, H-5’).

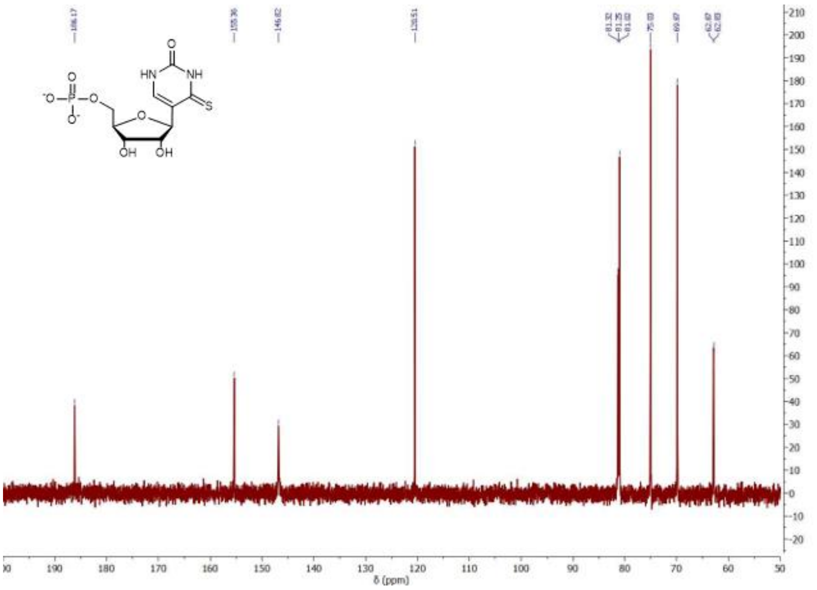

Supplementary Figure 17 ^{13}C NMR of 4-Thio-ΨMP (126 MHz, D_2O): δ 186.17 (s, 1C, C-4), 155.36 (s, 1C, C-2), 146.82 (s, 1C, C-6), 120.51 (s, 1C, C-5), 81.29 (d, J = 8.6 Hz, 1C, C-4’), 81.02 (s, 1C, C-1’), 75.03 (s, 1C, C-2’), 69.87 (s, 1C, C-3’), 62.85 (d, J = 4.8 Hz, 1C, C-5’).

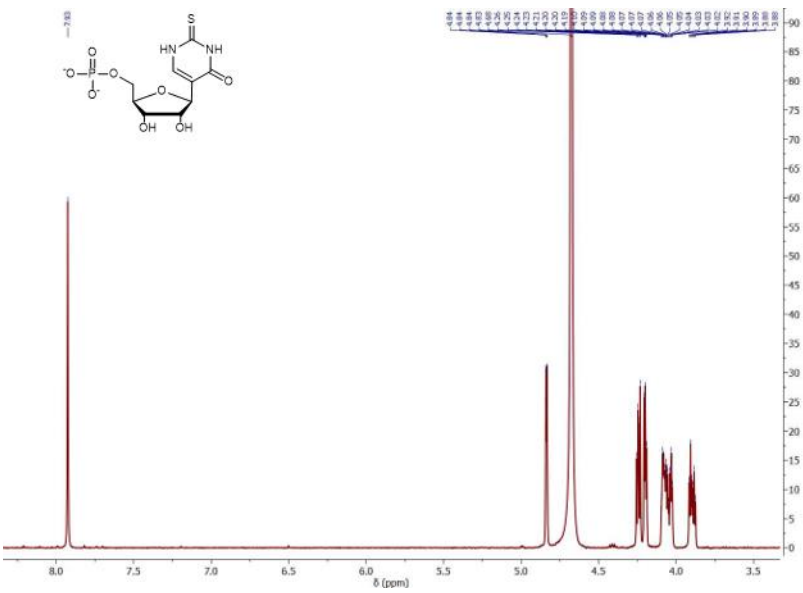

Supplementary Figure 18 ^{1}H NMR of 2-Thio-ΨMP (500 MHz, D_2O): δ 7.93 (s, 1H, H-6), 4.84 (dd, J = 3.4, 1.1 Hz, 1H, H-1'), 4.24 (dd, J = 7.0, 5.0 Hz, 1H, H-2'), 4.20 (dd, J = 5.0, 3.4 Hz, 1H, H-3'), 4.11 – 4.06 (m, 1H, H-4'), 4.06 – 4.01 (m, 1H, H-5'), 3.90 (dt, J = 11.7, 4.5 Hz, 1H, H-5').

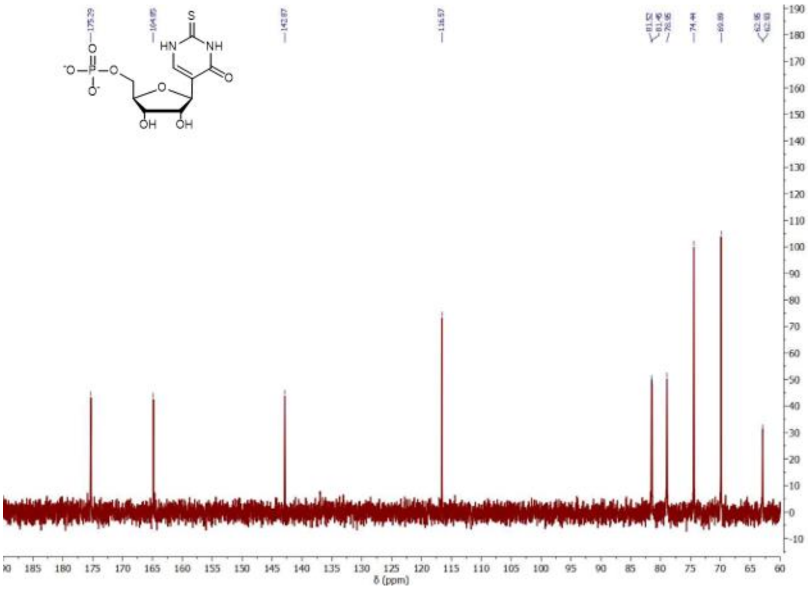

Supplementary Figure 19 ^{13}C NMR of 2-Thio-ΨMP (126 MHz, D_2O): δ 175.29 (s, 1C, C-4), 164.85 (s, 1C, C-2), 142.87 (s, 1C, C-6), 116.57 (s, 1C, C-5), 81.49 (d, J = 8.6 Hz, 1C, C-4'), 78.95 (s, 1C, C-1'), 74.44 (s, 1C, C-2'), 69.89(s, 1C, C-3'), 62.95 (d, J = 4.8 Hz, 1C, C-5').

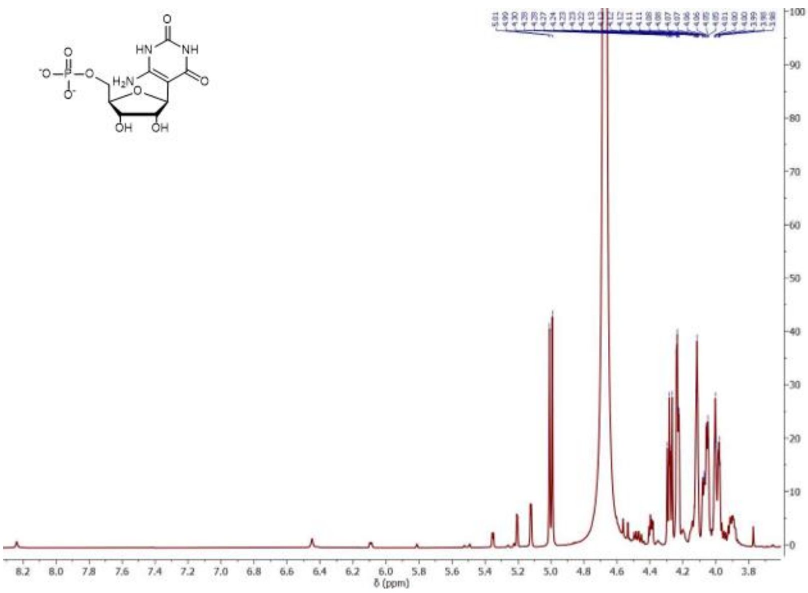

Supplementary Figure 20 ^{1}H NMR of 6-amino-ΨMP (500 MHz, D_2O): δ 5.00 (d, J = 8.8 Hz, 1H, H-1’), 4.28 (dd, J = 8.8, 5.8 Hz, 1H, H-2’), 4.23 (dd, J = 5.8, 2.5 Hz, 1H, H-3’), 4.13 (d, J = 2.0 Hz, 1H, H-4’), 3.99 (dd, J = 11.4, 5.3 Hz, 1H, H-5’), 3.90 (dd, J = 11.5, 2.2 Hz, 1H, H-5’). 30% of Rib5P impurity is present.

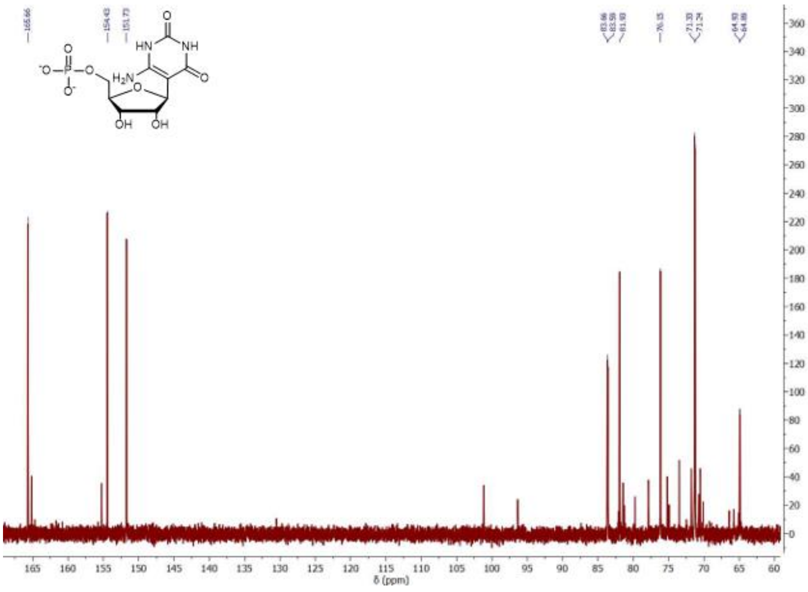

Supplementary Figure 21 ^{13}C NMR of 6-amino-ΨMP (126 MHz, D_2O): δ 165.64 (s, 1C, C-4), 154.42 (s, 1C, C-6), 151.71 (s, 1C, C-2), 83.62 (d, J = 8.8 Hz, 1C, C-4’), 81.93 (s, 1C, C-5), 76.15 (s, 1C, C-1’), 71.32 (s, 1C, C-2’), 71.24 (s, 1C, C-3’), 64.91 (d, J = 4.8 Hz, 1C, C-5’).

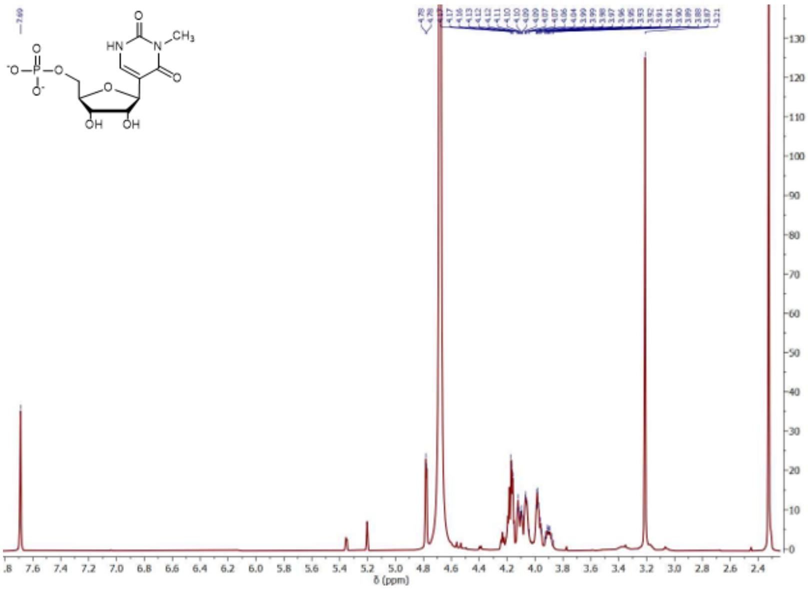

Supplementary Figure 22 ^{1}H NMR of 3-methyl-ΨMP (500 MHz, D_2O): δ 7.69 (s, 1H, H-6), 4.78 (d, *J* = 3.5 Hz, 1H, H-1’), 4.20 – 4.15 (m, 2H, H-2’ and H-3’), 4.11 (dq, *J* = 11.3, 2.9 Hz, 1H, H-4’), 4.08 – 4.04 (m, 1H, H-5’), 4.01 – 3.95 (m, 1H, H-5’) ,3.21 (s, 3H, C-7). 35% of Rib5P impurity and acetate are present.

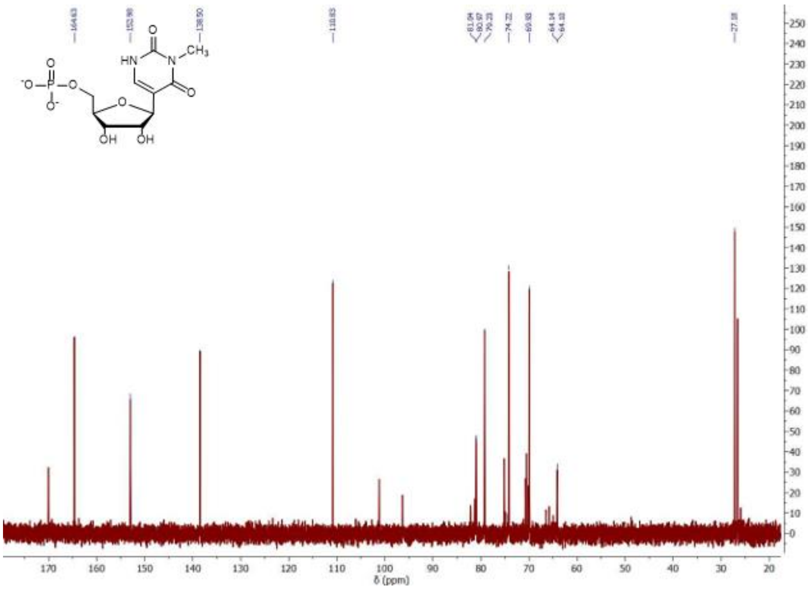

Supplementary Figure 23 ^{13}C NMR of 3-methyl-ΨMP (126 MHz, D_2O): δ 164.62 (s, 1C, C-4), 152.96 (s, 1C, C-2), 138.48 (s, 1C, C-6), 110.82 (s, 1C, C-5), 81.00 (d, J = 8.4 Hzs, 1C, C-4’), 79.23 (s, 1C, C-1’), 74.21 (s, 1C, C-2’), 69.93(s, 1C, C-3’), 64.12 (d, J = 4.9 Hz, 1C, C-5’), 27.18. (s, 1C, C-7).

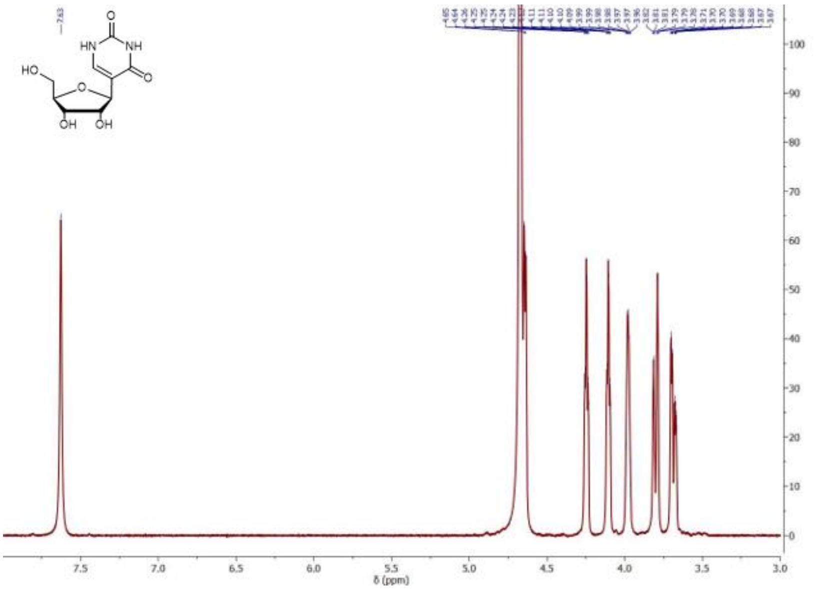

Supplementary Figure 24 ^{1}H NMR of Ψ (500 MHz, D_2O): δ 7.63 (s, 1H, H-6), 4.64 (dd, *J* = 5.6, 2.0 Hz, 1H, H-1’), 4.25 (td, *J* = 5.4, 2.0 Hz, 1H, H-2’), 4.10 (td, *J* = 5.4, 2.0 Hz, 1H, H-3’), 3.98 (m, *J* = 7.7, 4.9, 3.6 Hz, 1H, H-4’), 3.80 (dt, *J* = 12.9, 2.4 Hz, 1H, H-5’), 3.69 (ddd, *J* = 12.5, 4.8, 2.0 Hz, 1H, H-5’).

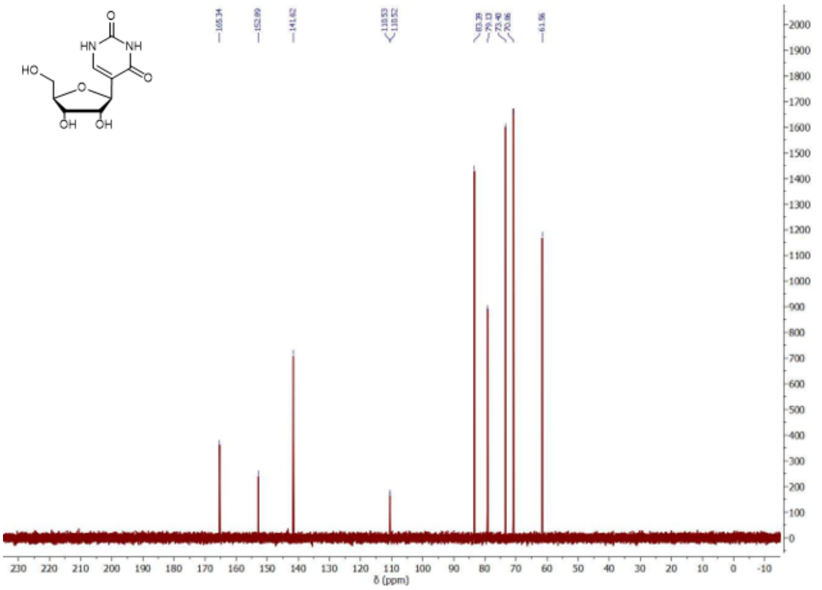

Supplementary Figure 25 ^{13}C NMR of Ψ (126 MHz, D_2O): δ 165.32(s, 1C, C-4), 152.87(s, 1C, C-2), 141.61 (s, 1C, C-6), 110.51 (s, 1C, C-5), 83.39 (s, 1C, C-4’), 79.12 (s, 1C, C-1’), 73.39(s, 1C, C-2’), 70.85(s, 1C, C-3’), 61.55(s, 1C, C-5’).

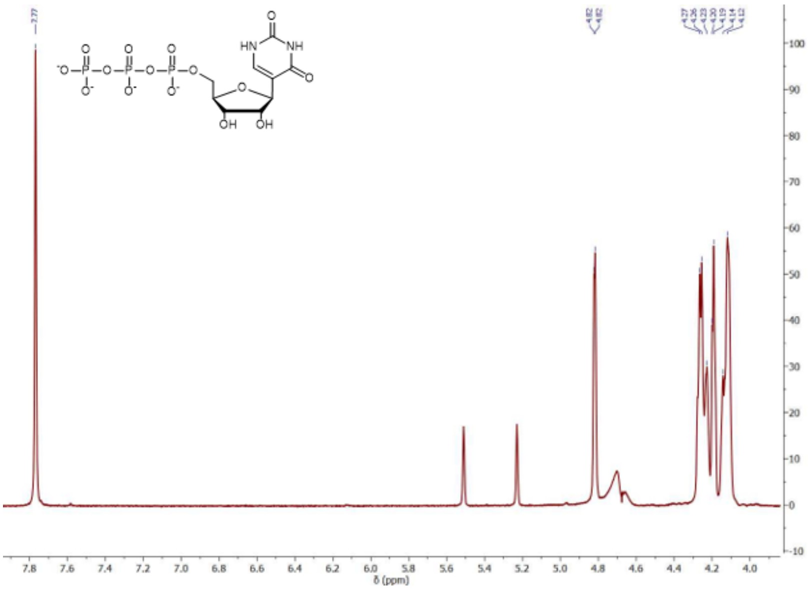

Supplementary Figure 26 ^{1}H NMR of ΨTP (500 MHz, D_2O): δ 7.77 (s, 1H, H-6), 4.82 (d, J = 4.0 Hz, 1H, H-1’), 4.30 – 4.08 (m, 5H, H-2’, H-3’, H-4’, H-5’). 20% of phosphoenolpyruvate impurity is present (δ 5.51, 5.23).

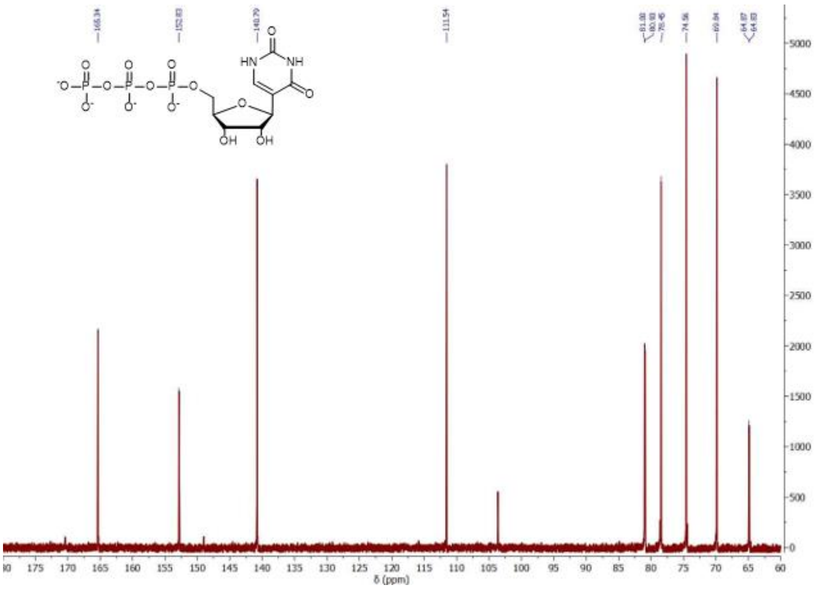

Supplementary Figure 27 ^{13}C NMR of ΨTP (126 MHz, D_2O): δ 165.32(s, 1C, C-4), 152.81 s, 1C, C-2), 140.78 (s, 1C, C-6), 111.53 (s, 1C, C-5), 81.00 (d, J = 9.0 Hz, 1C, C-4’), 78.60 (s, 1C, C-1’), 74.56 (s, 1C, C-2’), 69.83(s, 1C, C-3’), 64.87(d, J = 5.5 Hz, 1C, C-5’). 20 % PEP (δ 149.06, 103.6).

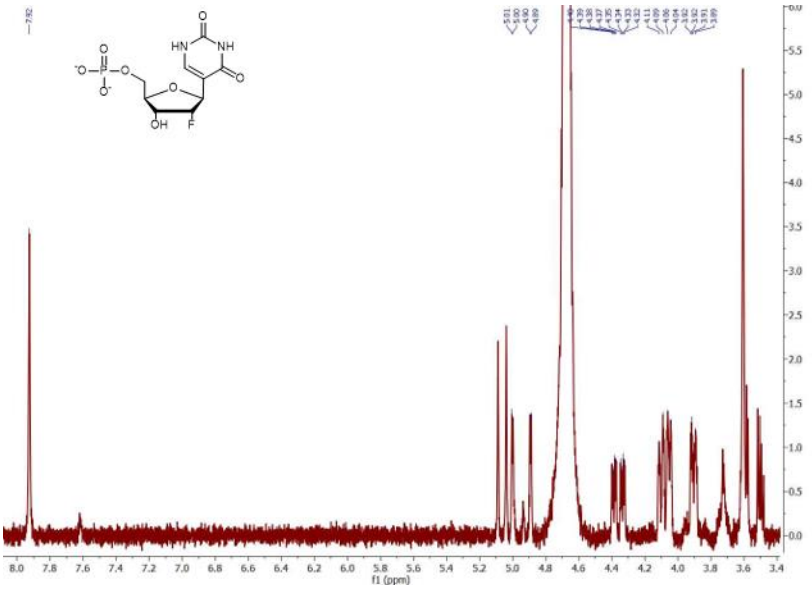

Supplementary Figure 28 ^{1}H NMR of 2-deoxy-2-fluoro-ΨMP (500 MHz, D_2O): δ 7.92 (s, 1H, H6), 4.95 (dd, J = 55.0, 3.9 Hz, 1H, H-1'), 4.36 (ddd, J = 26.1, 9.2, 3.9 Hz, 1H, H-2'), 4.10 (d, J = 11.8 Hz, 1H, H-5'), 4.05 (d, J = 9.5 Hz, 1H, H-3'), 3.91 (dd, J = 9.8, 5.4 Hz, 1H, H5'). Minor amounts of acetate, PEP and glycerol are present as impurities.

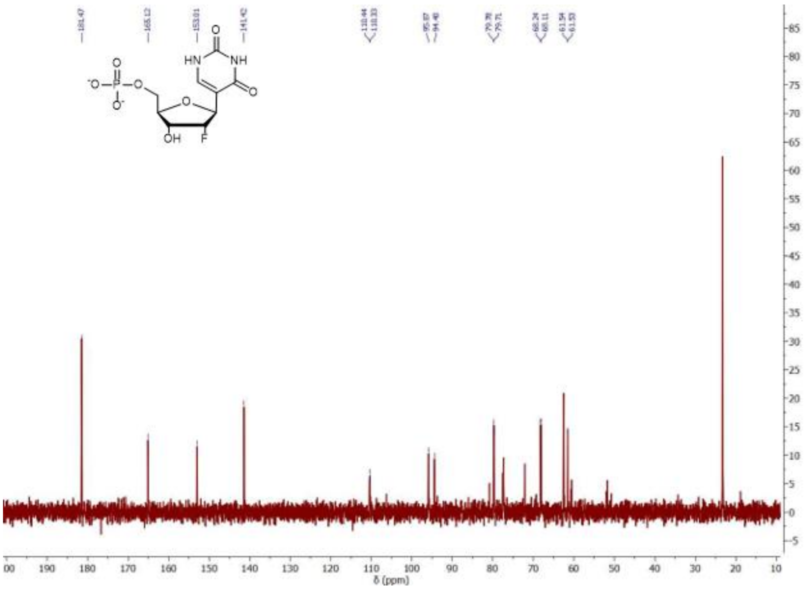

Supplementary Figure 29 ^{13}C NMR of 5-(β-D-2-deoxy-2-fluoro-ribopyranoside 5-phoshate)uracil (D-2F-Rib-ΨMP) (126 MHz, D_2O): δ 165.12 (s, 1C, C-4), 153.01(s, 1C, C-2), 141.42(s, 1C, C-6), 110.11 (s, 1C, C-5), 95.14 (d, J = 184.0 Hz, 1C, C-1'), 79.75 (d, J = 8.5 Hz, C-3'), 68.17 (d, J = 16.1 Hz, 1C, C-2'), 61.53(s, 1C, C-5').

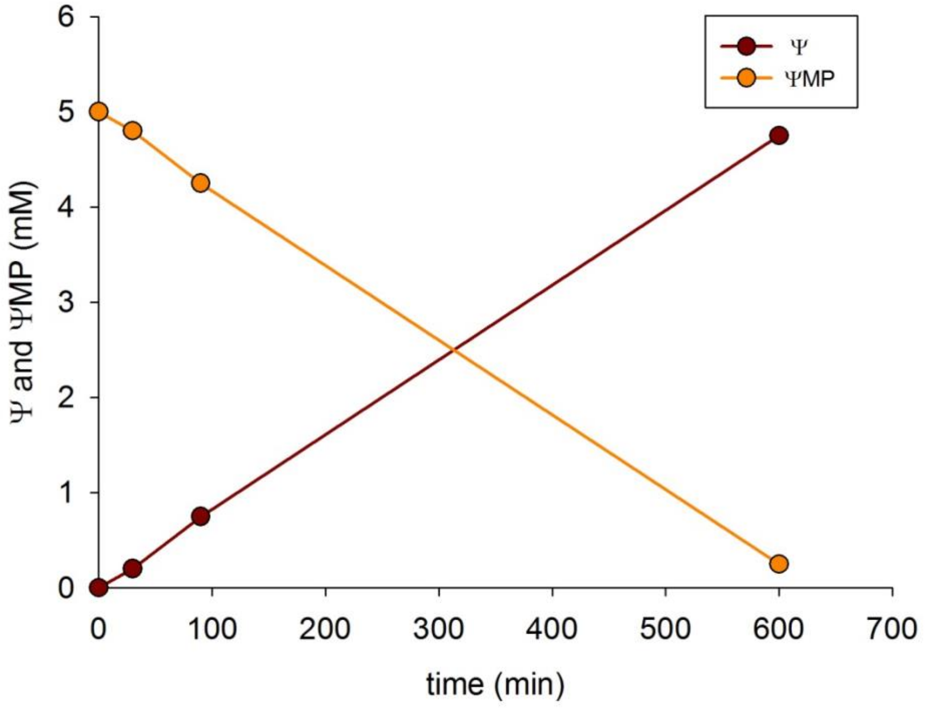

Supplementary Figure 30 Time course of the ΨMP dephosphorylation using calf intestine phosphatase (n = 1). For further experimental details and for the analytical procedures used, see the Methods section.

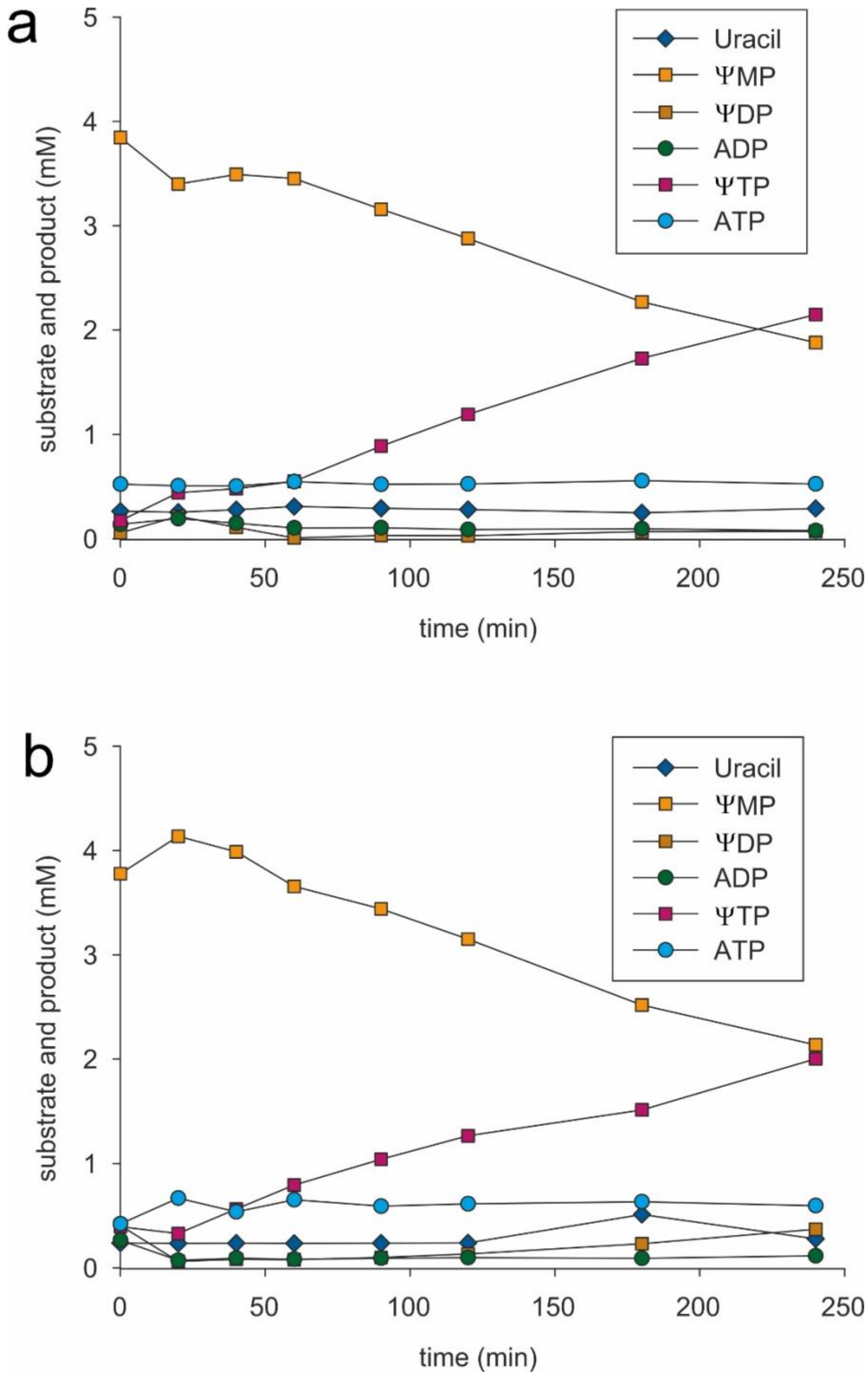

Supplementary Figure 31 Comparison of ΨMP phosphorylation with and without NDK. a) Time course of the CMPK-PK cascade for ΨTP synthesis (*n* = 1). b) Time course of the CMPK-PK-NDK cascade for ΨTP synthesis (*n* = 1). For further experimental details and for the analytical procedures used, see the Methods section.

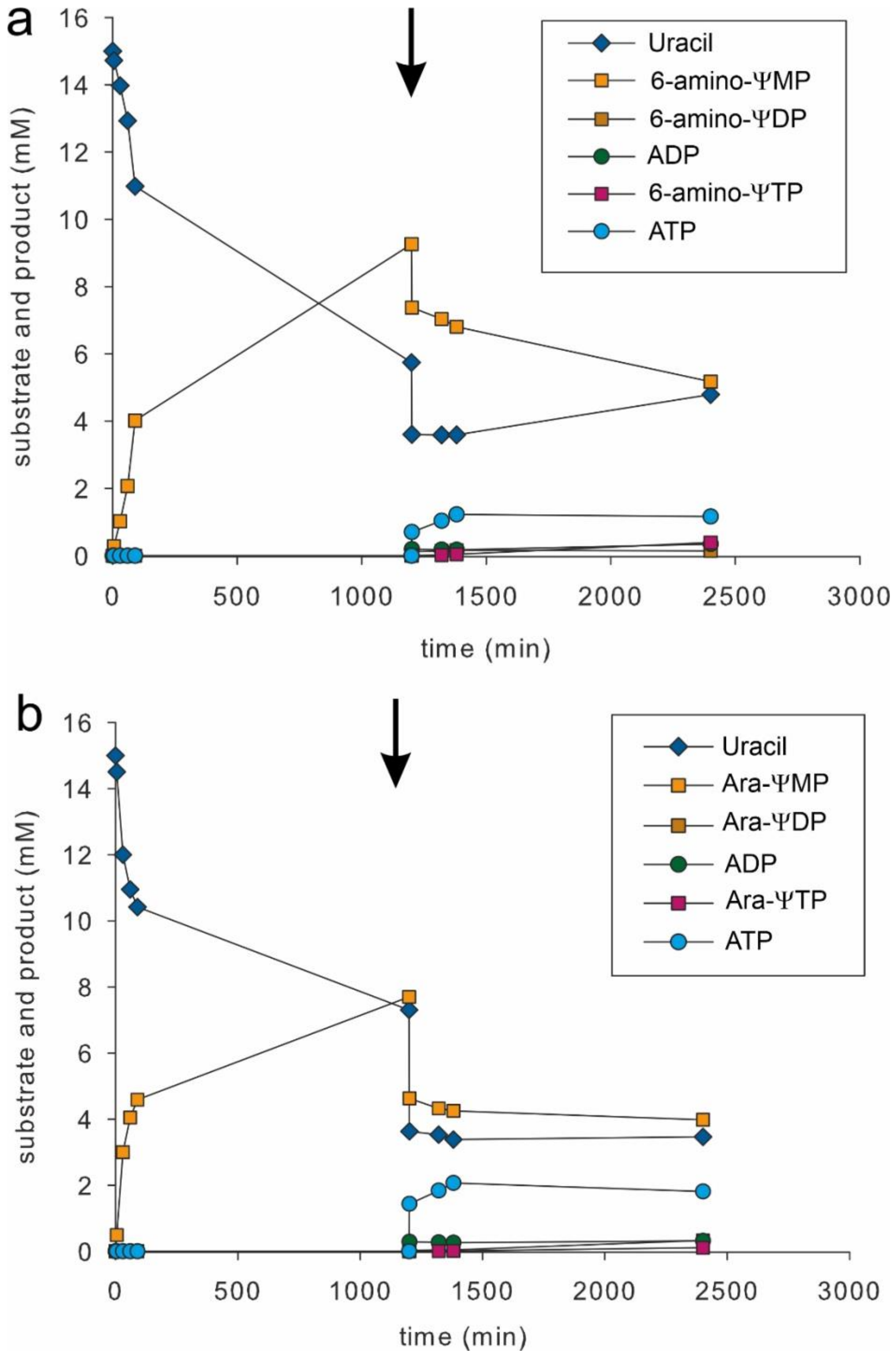

Supplementary Figure 32 One-pot multistep enzymatic synthesis of a) 6-amino-ΨTP (*n* = 1) and b) Ara-ΨTP (*n* = 1). Rib or Ara (100 mM) was phosphorylated by RbsK (15 µM for Rib, 60 µM for Ara) and pyruvate kinase (4 µM) using 100 mM PEP and 2 mM ATP as the phosphate donors (see Figure S4). ΨMP synthesis was started by the addition of 15 mM Ura, 1 mM $MnCl_2$, YeiN (90 µM) and 10 mM HEPES buffer (pH 7.0), resulting in a concentration of 25 mM pentose 5-phosphate (time point zero a, b). The arrow indicates the start of the phosphorylation reaction by addition of 15 mM PEP, 15 µM CMPK, and 1 mM ATP, resulting in a volume increase of 33 %. For further experimental details and for the analytical procedures used, see the Methods section.

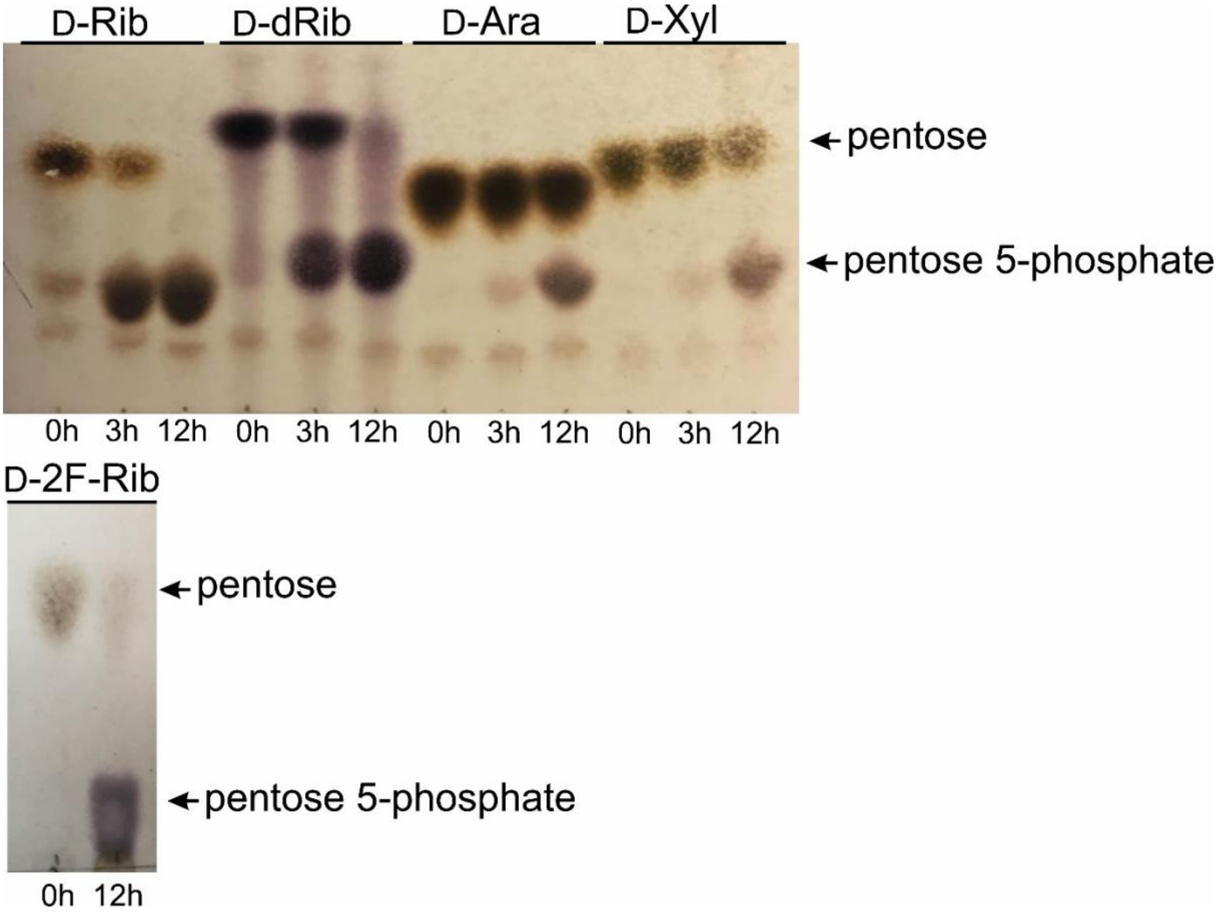

Supplementary Figure 33 Representative TLC analysis of pentose phosphate formation by RbsK. Pentose (100 mM) was incubated with RbsK (15 µM for D-Rib, 30 µM for D-dRib and 60 µM for D-Ara, D-Xyl and D-2F-Rib), 0.4 µM PK, 100 mM PEP and 2 mM ATP in 10 mM Hepes, pH 7.0, supplemented with 1 mM $MgCl_2$. Sample (1 µL) were spotted onto a TLC plate (Merk, Darmstadt, Germany) and run using a mobile phase consisting of 2-BuOH: AcOH: H_2O (2:1:1). Plates were sprayed with detection solution (0.5 g thymol, 95 mL EtOH and 5 mL H_2SO_4) and developed by heat treatment. (n=1)

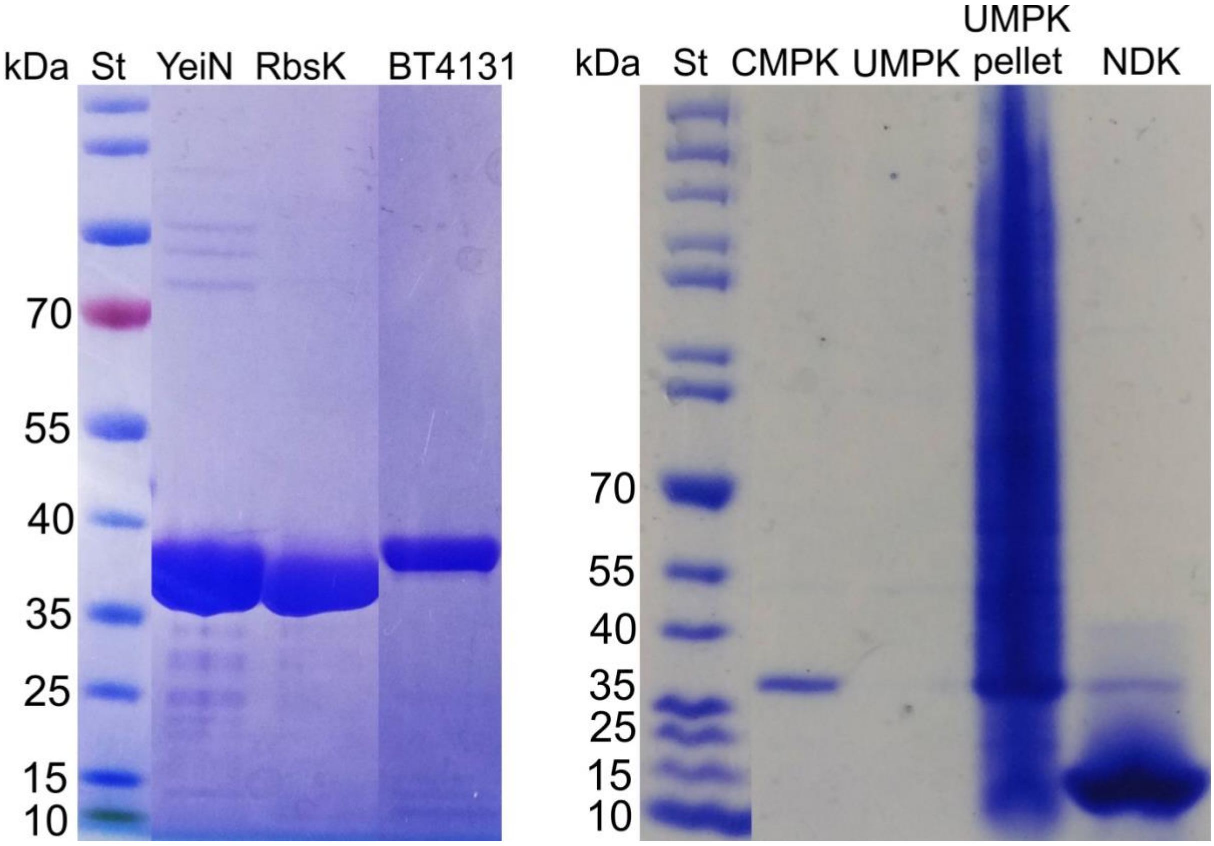

Supplementary Figure 34 SDS polyacrylamide gel showing the purity of the enzyme preparations used. St: protein size standard, YeiN: β-pseudouridine 5’-phosphate glycosidase, RbsK: ribokinase, BT4131: pentose-5-phosphate phosphatase, CMPK: cytidine monophosphate kinase, UMPK: uridine monophosphate kinase, NDK: nucleotide diphosphate kinase. The UMPK pellet is the insoluble protein obtained from *E. coli* expression culture.

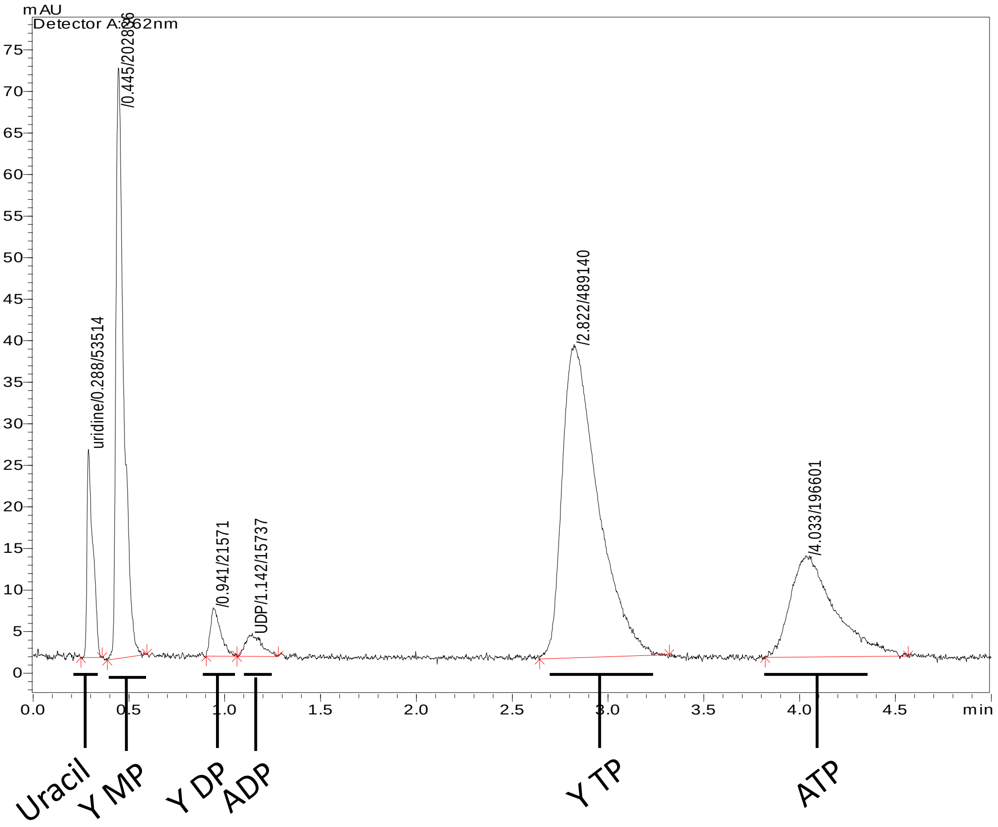

Supplementary Figure 35 HPLC trace of reference substances used and their separation: uracil (0.3 min), ΨMP (0.4 min), ΨDP (0.9 min), ADP (1.1 min), ΨTP (2.7 min) and ATP (4 min). For further details, see the Methods section.

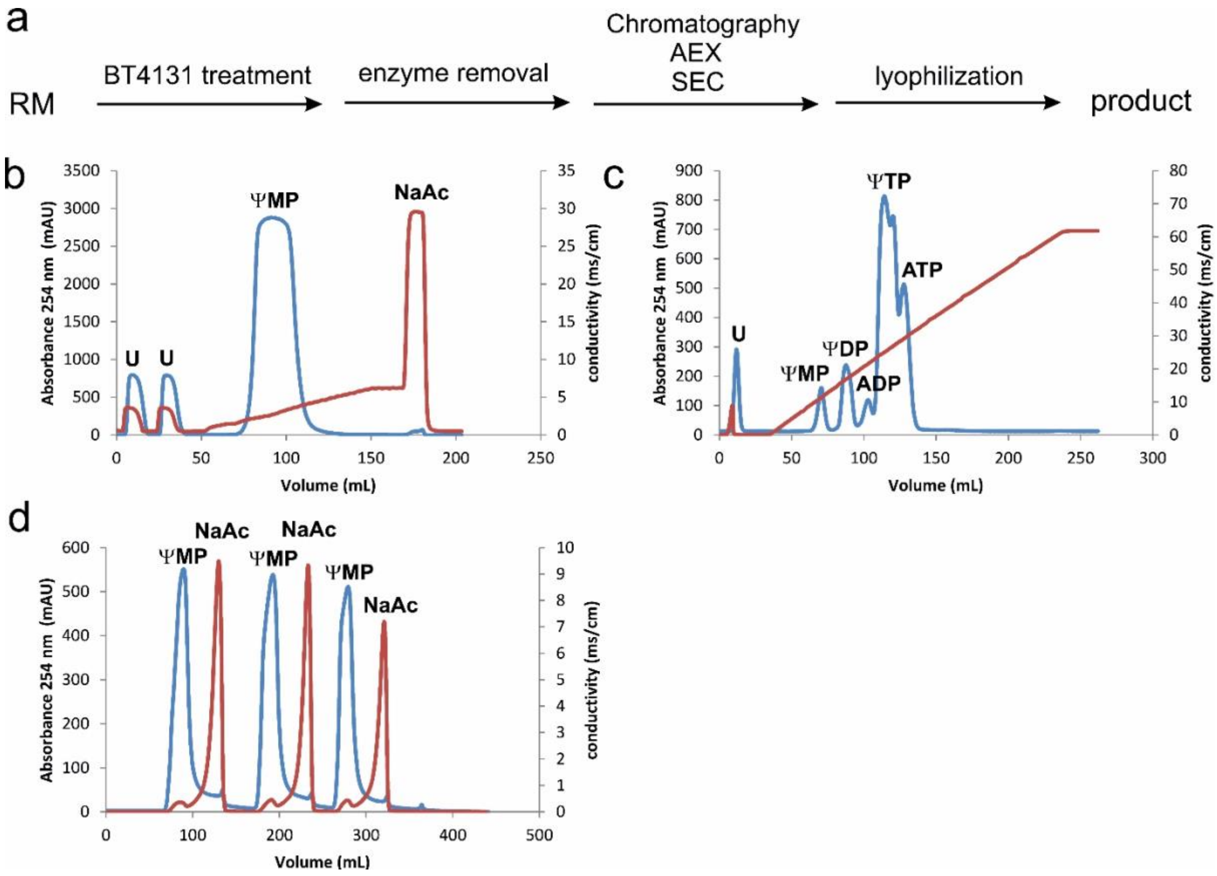

Supplementary Figure 36 Product isolation. a) Scheme of the purification route used. b-c) Typical chromatography traces of the AEX purification of (b) ΨMP and (c) ΨTP or the size exclusion chromatography (SEC) purification of ΨMP. For further experimental details, see the Methods section.

Supplementary References

1. Huang, S., Mahanta, N., Begley, T. P. & Ealick, S. E. Pseudouridine monophosphate glycosidase: a new glycosidase mechanism. *Biochemistry* **51**, 9245–9255 (2012).
2. Oja, T. *et al.* Structural basis for C-ribosylation in the alnumycin a biosynthetic pathway. *Proc. Natl. Acad. Sci. U. S. A.* **110**, 1291–1296 (2013).
3. Blauenburg, B., Oja, T., Klika, K. D. & Metsä-Ketelä, M. Chemoenzymatic synthesis of novel C-ribosylated naphthoquinones. *ACS Chem. Biol.* **8**, 2377–2382 (2013).
